# The Eosinophilia of Hydatid Disease

**Published:** 1908-05-02

**Authors:** 


### PATHOLOGY.

### THE EOSINOPHILIA OF HYDATID DISEASE.

Hydatid disease is not very common in England, though it is no rarity in other places, notably in certain parts of Australia. The very fact that the complaint is not met with every day here makes its diagnosis a matter of some difficulty. Everyone of us is confronted, sooner or later, with the problem whether a given lump in the liver, the peritoneum, or elsewhere is a malignant neoplasm, a gumma, or a hydatid cyst, and the question is often settled only by an operation. Every point that can assist in the diagnosis is therefore valuable, and one such point is the differential leucocyte count.

Not a few cases of hydatid disease exhibit eosinophilia. Unfortunately, like almost every other clinical method, it is not pathognomonic—that is to say, there are many exceptions to the general rule. In some cases the coarsely granular eosinophile cells may amount to 10 or 15 per cent. of all the leucocytes in the blood, in which case there is strong confirmation of the suspected hydatid cyst; on the other hand, such a cyst may be present, and yet the proportion may be only 2 per cent. It is of extreme interest to know why there should be such variations in different cases; and a good deal of light has recently been thrown upon the subject by the researches of Boidin, Fiessinger, and others.

The evidence that these observers have brought forward goes to show that the eosinophile reaction in these patients begins in the immediate neighbourhood of the cyst wall. In a case in which the cyst is quiescent the reaction spreads no further, and the blood in the general circulation exhibits no eosinophilia. If, however, the cyst is not quiescent the eosinophile reaction spreads more widely, and a variable degree of general eosinophilia results. It would follow, therefore, that if a patient had a lump which might be a hydatid cyst, and if the lump were causing no symptoms, eosinophilia would not be expected, and the absence of eosinophilia would not exclude the diagnosis of hydatid disease. If, on the other hand, the lump in question were causing pain, pyrexia, or other evidence of its activity, and there were no eosinophilia, the argument would be against hydatid disease.

The work upon which the authors base these conclusions is interesting. They cut a series of sections of the walls and surroundings of the hydatid cysts obtained from two patients who were operated upon by M. Quenu. In the first case the cyst contained air in addition to turbid but acellular fluid, and the patient had exhibited no eosinophilia the day before the operation. In the second case the cyst was an old one, enclosing a small quantity of almost clear, acellular fluid, and some daughter cysts, of which many were undergoing degeneration.

### Histology of the First Cyst Wall.

The cyst was bulging up the hepatic parietes. Macroscopically it seemed to be formed of very vascular granulation tissue. The hydatid membrane itself could not be made out, for it was entirely replaced by granulation tissue. Sections were prepared and four zones could be made out.

Zone 1.—Close to the internal aspect of the cyst there was a thin stratum which had no organised structure. It stained deeply with eosine, and here and there it exhibited collections of small round cells—miliary abscesses. This zone represented the hydatid membrane in a state of necrobiosis.

Zone 2.—Immediately outside Zone 1 there was a layer of loose immature connective tissue, in which there were numbers of capillaries containing blood, and other capillaries budding off from these but not yet pervious to the blood-stream. In the meshes of this tissue there were quantities of embryonic cells. Amongst the latter lymphocytes predominated, but they were less closely packed than in ordinary lymphoid tissue. There were also fairly large numbers of eosinophile cells, some of them polymorphonuclear; others with bilobed and compressed nuclei; and others again quite small with dense round deeply stained single nuclei. Besides the lymphocytes and the eosinophile cells there were a good many red blood corpuscles, some ordinary polymorphonuclear leucocytes, and very small numbers of macrophages. No mast cells were seen. The eosinophile cells were almost entirely in the connective tissue spaces; very exceptionally they occupied the lumen of capillary vessels.

Zone 3.—Around the above was a zone of denser connective tissue, with close-set collagenous fibrils and cells with elongated nuclei. Amongst these were a few arterioles, capillaries engorged with blood, and bile canaliculi, the cells lining the latter having nuclei which stained very clearly. No collections of small round cells were present in this zone, and there was no interstitial eosinophilia.

Zone 4.—The outermost zone of all exhibited almost normal hepatic tissue, or peritoneum, as the case might be, without any sign of eosinophile cells.

### Histology of the Cyst Wall in the Second Case.

This cyst wall was extremely dense, thick, and fibrous. Its internal zone was formed of close-set fibrous tissue, without any leucocytic infiltration. A little outside this, in the midst of dense connective tissue, masses of embryonic cells were seen, but none of these exhibited eosinophilia. Between this and the normal hepatic tissue there was a zone of fibrous tissue in which many blood-vessels and bile canaliculi were present. In this layer there were a good many lymphocytes, but only a very occasional eosinophile cell; no eosinophile leucocytes could be detected within the blood-vessels.

---

The histological examination of the first cyst wall suggests that the eosinophile cells are derived from others which become loaded with granules at the same time that the nucleus, from being round, changes into first a bilobed and then into a polymorphic shape. It seems more than likely that this change results from reaction to toxins from the cyst; and the fact that small mononuclear lymphocytes may thus become transformed into polymorphonuclear eosinophile leucocytes has been demonstrated by Dominici.

The eosinophile transformation being restricted to the inner zones of the cyst wall explains why the patient's blood exhibited no general eosinophilia.

The histology of the second cyst wall explains still better why there was no general eosinophilia in that case if, as one may suppose, the general eosinophilia is a result of extension from a local eosinophilic reaction. There was none of the latter at all in this case. Several observers have noted that involuting hydatid cysts give rise to no eosinophilia. This absence of local reaction may be explained by the extent of the peri-cystic sclerosis, which had in a sense strangled the masses of embryonic cells whence are derived the eosinophile corpuscles, and which had also formed an insuperable barrier against toxic substances from the cyst. It is probable, moreover, that the toxicity of the fluid in an involuting cyst is comparatively slight. Hence the restricted value that eosinophilia possesses as a clinical means of differentiating between hydatid disease on the one hand and other tumours which may simulate it upon the other.

### Morphine Contracts.

Owing to the uncertain position of the market for opium alkaloids occasioned by the unsettled state of the market in the raw material, buyers of morphine have hesitated to make contracts in view of the possibility of a fall in the value of this drug. Consequently manufacturers have been making comparatively few new contracts and have had to be content with the small orders submitted by purchasers who for the most part have simply been buying to suit their immediate requirements. With the object of encouraging buyers, makers have altered their conditions of sale by introducing a reduction clause, which is in the following terms: "In event of our price being reduced during currency of this contract any deliveries the buyer may call up will be invoiced at the lower price fixed by us while such reduction remains in force. And should our price revert to the original or higher figure than price of this contract, the original contract price becomes again operative. Contracts existing at this date will participate in the benefit of this clause until expiry of the delivery period." While this new condition of sale will have no direct influence on small purchases, hospitals buying on contract terms should benefit by the innovation. Codeine, which is also sold subject to a reduction clause, has just been reduced in price by 8d. per oz. As regards opium, it is impossible, owing to the conflicting nature of the reports from Smyrna, to make any reliable forecast as to the future of this drug; in the meantime, however, the tendency is to easier rather than to firmer prices.

